# Do Psychological Resilience and Emotional Intelligence Vary Among Stress Profiles in University Students? A Latent Profile Analysis

**DOI:** 10.3389/fpsyg.2021.788506

**Published:** 2022-01-28

**Authors:** Büşra Kökçam, Coşkun Arslan, Zeliha Traş

**Affiliations:** Department of Guidance and Psychological Counseling, Necmettin Erbakan University, Konya, Turkey

**Keywords:** stress, emotional intelligence, psychological resilience, university students, latent profile analysis

## Abstract

The coronavirus/COVID-19 pandemic has brought about significant changes in the lifestyle of students. However, despite an extensive study of students’ life stress using a non-comprehensive scale and variable-centered approach, it has been little studied with a comprehensive scale and person-centered approach. Using the Student-Life Stress Inventory-revised (SSI-R), we analyzed students’ latent stress profiles and examined differences in psychological resilience and emotional intelligence by comparing stress profiles from a sample of 418 undergraduate and graduate students (aged 18–36) in various departments of eight universities in Turkey. We identified five distinct stress profiles, defined as an extremely low stress group (ELSG), a low stress group (LSG), a medium stress group (MSG), a high stress group (HSG), and an extremely high stress group (EHSG). We found that (1) MSG and HSG were similar in terms of emotional intelligence, resilience, and possession of high standards, and they reported higher levels of physiological, emotional, and behavioral reactions than ELSG and LSG; (2) MSG felt more pressure than HSG; (3) ELSG reported higher levels of emotional intelligence (wellbeing, self-control, and emotionality) than others. Also, EHSG reported lower levels of emotional intelligence (specifically self-control) than others; (4) whereas resilience was highly positively correlated to wellbeing, resilience and wellbeing were moderately negatively correlated to stress. Extremely low stress group and LSG reported higher levels of resilience than others. Medium stress group, HSG, and EHSG did not differ with regard to resilience and wellbeing. Our results suggest that, university students are able to maintain their functionality by coping up with stress in some ways, no matter how stressful they are. These findings are discussed in relation to the relevant literature.

## Introduction

After the outbreak of coronavirus/COVID-19, in-person learning has been suspended in schools affiliated to the Ministry of National Education and universities in Turkey as of March 14, 2019, and the educational process has been resumed with distance learning systems (Eken et al., 2020). Although online education has gradually increased since the pre-pandemic period, the majority of students were not familiar with distance learning systems at the beginning of the pandemic period. This situation has become even more challenging when combined with problematic home environments and the lack of access to academic resources. Students who resume their learning at home have experienced a distraction due to additional responsibilities brought by other family members and sudden changes in lifestyle. Difficulty focusing on learning, performance anxiety in the online classroom environment, and uncertainties about how to proceed have led students to experience significant academic stress (Aslan et al., 2020; Husky et al., 2020; Li et al., 2020; Moawad, 2020; Patsali et al., 2020; Son et al., 2020; Clabaugh et al., 2021; Grace et al., 2021). Although students are faced with similar situations, there are differences in the degree and type of their reactions. Lazarus and Folkman (1984) examined the way individuals interpret stressful situations cognitively in primary and secondary appraisals. In primary appraisals, a person assesses whether the situation is (a) irrelevant, (b) benign-positive, or (c) stressful. If the situation has no effect on a person’s wellbeing, an irrelevant category is selected. If the situation includes harm/loss, threat, and challenge, the stressful category is selected. While negative emotions such as fear and anxiety are at the center of threat appraisals, challenge appraisals focus on the potential for gain or growth, and they are characterized by pleasurable emotions such as eagerness, excitement, perseverance, hope, and confidence. Mostly, these situations are intertwined. In secondary appraisals, a person decides whether they have resources and effective strategies to cope with a stressful situation (pp. 31–33).

Although stress is a widely studied topic, it involves a complex relationship between overlapping and interacting of a large number of stressors and reactions that produce multiple behavior. For instance, physiological reactions may also be emotional or behavioral reactions (Gadzella, 1994). Because stress has a complex structure, which includes many stressors and reactions, necessary to examine it in terms of these variables. However, to deal with it comprehensively, most of the scales used in numerous studies on stress do not seem to be sufficient. Perceived Stress Scale (PSS-10) and Depression, Anxiety, and Stress Scale (DASS), which are the frequently used scales, measure responses to stress but provide a greatly limited measure of stressors (Cohen et al., 1994; Lovibond and Lovibond, 1996; Lee, 2012). Student-Life Stress Inventory developed by Gadzella for university students and later revised, includes five stress categories (frustrations, conflicts, pressures, changes, and self-imposed) and four reactions to the categories of stressors (physiological, emotional, behavioral, and cognitive appraisal) (Gadzella, 1994; Baloğlu and Bardakcı, 2010).

The Frustrations category refers to the frustrations that occur due to delays, daily hassles to reach goals, the lack of resources (e.g., money and books), failures to accomplish goals, feelings of being a social outcast, interpersonal relationship problems, and denial of opportunities in spite of one’s qualifications. The Conflicts category points out the conflicts that came out of having two or more desirable and undesirable alternatives and when a goal had both positive and negative alternatives. The Pressures category indicates the pressures that are due to competition, deadlines, work overload, responsibilities, and expectations (e.g., interpersonal relationships and work). The Changes category refers to the stress produced by unpleasant or excessive changes that are disruptive to the participant’s life/goals. The Self-Imposed category points out the stress that came out of the participant’s competitive attitude, desire to be noticed and loved by all, his/her common anxiety, perfectionist, and procrastinative tendencies (Gadzella, 1994). In the reactions to stressors’ section, the Physiological Reactions category indicates responses such as sweating, stuttering, hyperventilation, trembling, exhaustion, skin itching, weight loss/gain, and headaches. The Emotional Reactions category refers to fear, anxiety, worry, anger, guilt, depression, and grief. The Behavioral Reaction category points out crying, drug use, smoking, and to be isolated oneself from others. Finally, the Cognitive Reactions category indicates the participant’s ability to think about and analyze stressful situations and use the most effective strategies (Gadzella, 1994). According to the study by Bell et al. (2012) on academicians, wellbeing did not decrease as the workload and pressure increased. A lot of studies have shown that some people achieve the best performance under pressure. These people see a stressful situation as an exciting experience (see Ferrari et al., 2009; Grunschel et al., 2013). However, an overwhelming amount of research provides evidence for a negative relationship between stress and wellbeing and for a positive relationship between wellbeing and resilience (Durand-Bush et al., 2015; Li and Hasson, 2020). Fletcher and Sarkar (2013) stated that psychological resilience is the most important and prominent resource in coping with stress.

Luthar (2015) explained psychological resilience based on two basic concepts: adversity and positive adaptation. According to Luthar and Cicchetti (2000), adversity includes negative life circumstances associated with adjustment difficulties (p. 858). Davis et al. (2009) stated that adversity consists of modest disruptions rather than major disasters faced by people in their daily lives. On the other hand, the positive adaptation has been defined as manifesting social competence behaviorally, or meeting stage-salient developmental tasks successfully (Luthar and Cicchetti, 2000, p. 858) or displaying symptoms related to internal wellbeing (Masten and Obradović, 2006, p. 15). Wagnild and Young (1993) defined resilience as a positive personality characteristic that increases emotional stamina, courage, and adaptability in the face of life difficulties. Resilience consists of five components: equanimity, perseverance, self-reliance, meaningfulness, and existential aloneness. Equanimity refers to having a balanced perspective on life, so that one faces stressful situations with calmness and not with heightened reactivity. Despite its adversity or discouragement, perseverance refers to a person’s willingness to keep going and to strive to reconstruct one’s life. Those who are self-reliant recognize and rely on their personal strengths, capabilities, and past successes. Existential loneliness is defined as the feeling of freedom and authenticity in which each person’s life path is unique, some moments are shared with others, but at other times the person continues alone (Wagnild, 2009). Kobasa (1979) remarked that individuals with high psychological resilience see changes and stress in their lives as opportunities, and rely on themselves (belief in their values, goals, and capacities) instead of alienation from themselves. Despite their difficulties, they are meaning-oriented rather than a nihilism, internal rather than external locus of control, they are aware of the limits of what they can control, and they adopt an active approach instead of a passive approach to solving the problem. Rutter (1985) stated that these people are securely attached to others and have personal or collective goals; see difficulties as a tool to become stronger; and have a success in their personal history, high self-efficacy, and a developed sense of humor. According to the study by Lyons (1991), people with high psychological resilience have high levels of patience, negative effect tolerance, and adaptability in the face of changes. These traits reflect important internal mechanisms to individuals that influence their resilience to stressors. Lots of studies have reported a moderately significant negative correlation between stress and resilience (Durand-Bush et al., 2015; Sanderson and Brewer, 2017; Cooper et al., 2020; Kim et al., 2021; Labrague, 2021). However, in some of these studies, although students reported a high level of stress, their levels of resilience and psychological wellbeing were found to be moderate to high (Durand-Bush et al., 2015; Labrague, 2021).

Troy and Mauss (2011) remarked that due to the presence of highly emotional stressful events, people’s ability to regulate their emotions is determinative on their psychological resilience and wellbeing. Salovey et al. (1999) theorized that people with high emotional intelligence are able to perceive and appraise their emotions accurately, know how and when to express their emotions, and can better cope with the emotional demands of stressful encounters because they can regulate their moods effectively (p. 161). Emotional intelligence is structurally defined as two different models, ability and mixed (traits with abilities) (Mayer et al., 2000). Emotional intelligence, as a self-reported personality trait, indicates how emotionally competent one feels. However, emotional intelligence as the ability based on the maximum performance test indicates the cognitive-emotional ability of a person (Petrides, 2009). Petrides et al. (2007) contended that a person’s unique emotional experience cannot be measured only through an objective maximum performance test and that self-report measurement tools are needed for assessing emotional intelligence as a personality trait. In the Trait Emotional Intelligence Questionnaire (TEIQue) developed by Petrides, emotional intelligence as a personality trait has 15 facets: adaptability/flexibility, assertiveness, recognizing one’s own and others’ emotions, expressing one’s emotions, regulating their emotions, managing others’ emotions (capacity to influence others’ emotions), low impulsivity, capacity to maintain personal relationships, self-esteem, self-motivation, social awareness, trait empathy, trait happiness and optimism, and stress management. These facets are grouped into four factors: emotionality, sociability, wellbeing, and self-control. The emotionality factor includes trait empathy, emotion perception, emotion expression, and the maintenance of personal relationships. The sociability factor includes emotion management, assertiveness, and social awareness. In sociability, the focus is on the social context outside one’s family or close friends. People with a high level of sociability are good listeners and confidently communicate with people from different backgrounds. The factor of wellbeing includes self-esteem, trait happiness, and optimism, and the factor of self-control includes emotion regulation, stress management, low impulsivity, adaptability/flexibility, and self-motivation (Petrides and Furnham, 2001; Petrides, 2009). Lots of studies have shown that people with high emotional intelligence cope better with stress (Cejudo, 2016; Enns et al., 2018; Trigueros et al., 2020; Mérida-López and Extremera, 2021; Toriello et al., 2021). However, some studies have reported that some people with high levels of emotional perception and/or emotional sensitivity to angry expressions experience a high level of anxiety (Gutiérrez-García and Calvo, 2017; Schwab and Schienle, 2017; Cui et al., 2021). Also, Ciarrochi et al. (2002) reported that people with high emotional perception of their own emotions are more affected by stress and express higher levels of depression, hopelessness, and suicidal thoughts (p. 205).

The purpose of this study is to examine the stress profiles of university students in terms of psychological resilience and emotional intelligence (emotionality, sociability, wellbeing, and self-control), which are the variables related to wellbeing. Also, it was examined whether some counterintuitive findings referred earlier could be explained based on stress profiles. As far as we know, there are no other studies examining the stress of university students by person-centered analysis, except a recent stress profile analysis study on engineering students (Perkins et al., 2021). The profile analysis groups people based on the similarity of their response patterns, unlike a variable-centered approach that focuses on constructs. In this way, insights can be gained to understand people’s attitudes, beliefs, and mindsets. Thus, stress profiles can help tailor interventions to reduce stress as well as to enhance an understanding of the complex mechanism of stress in higher education.

Our research questions (RQ) are: (RQ1) How many homogenous profiles would emerge according to Student-Life Stress Inventory-revised (SSI-R)? (RQ2) To what extent do these profiles differ across the nine categories? (frustrations, conflicts, pressures, changes, self-imposed, physiological reactions, emotional reactions, behavioral reactions, and cognitive reactions)? (RQ3) To what extent do these profiles differ across psychological resilience and emotional intelligence (emotionality, sociability, wellbeing, and self-control)?

## Materials and Methods

### Participants and Data Collection Procedure

After excluding ten respondents with missing responses and three respondents based on extreme outlier patterns, the final sample of this study consisted of 418 participants. The participants’ age was 18–36 years (*M* = 21.21, SD = 2.93). Of these participants, 272 (65%) were female and 140 (34%) were male. Six of the participants (1%) did not want to indicate their gender. The majority of participants were undergraduate students (359, 86%) from various faculties (dentistry, education, arts and sciences, engineering, theology, medicine, economics, administrative sciences, etc.), 59 participants (14%) are graduate students from various institutes (health science, social sciences, education sciences, and science and technology). In Turkey, the length of medical school undergraduate program is 6 years, and the length of dentistry school undergraduate program is 5 years. Among the participants, 78 (19%) were 1st year, 91 (22%) 2nd year, 96 (23%) 3rd year, 59 (14%) 4th year, 22 (5%) 5th year, 13 (3%) and 6th year, 59 (14%) were students enrolled in master’s and/or doctoral degree educational programs.

The data were collected in June and July 2021. Given the ongoing restrictions on in-person data gathering, the questionnaires were coded on the online survey platform Google Forms. The link to the survey was sent to academic advisors and/or student representatives in various faculties/institutes at eight universities in Turkey and asked to be shared on online platforms for classroom groups. In the first page of the online survey, it was stated that the data obtained would only be used for scientific articles and participation in the study was completely voluntary and the email address of the first author was provided for contact in case of doubts or need. Those who agreed to participate were asked to click the “I approve” check box in the form and were redirected to the online questionnaire. Anonymity was ensured, and any personal identification, such as IP address, and email IDs were not requested. All procedures followed were in accordance with the standards of the Helsinki Declaration. The research described in this article was approved by the Scientific Research and Publication Ethics Committee of Social and Human Sciences of Uşak University (ref no. 2021–122).

### Measures

#### Student-Life Stress Inventory-Revised

Students’ stress was measured using 53 items and two sections (stressors and reactions to stress), which have rated on a 5-point Likert-type scale (from 1 = “*never*” to 5 = “*most of the time*”). The stressors section consists of five categories such as frustrations, conflicts, pressures, changes, and self-imposed, and the reactions to the stressors section consist of four categories such as physiological reactions, emotional reactions, behavioral reactions, and cognitive reactions. The total score is obtained by summing the scores from the items in the two sections. High scores indicate a high level of stress. The scale was adapted into Turkish by Baloğlu and Bardakcı (2010). In this study, Cronbach’s alpha was 0.94 for the total scale and the nine categories showed appropriate Cronbach’s alphas ranging between 0.86 (conflicts and physiological reactions) and 0.61 (self-imposed).

In the seven previous studies using the Student-Life Stress Inventory, college students were asked to evaluate their own stress levels (mild, moderate, and severe), and the average scores of nine categories of the grouped students were calculated. The mean values of total stress score were in the range between 109.6 and 177.6 (see Gadzella, 2004; Baloğlu and Bardakcı, 2010; Gadzella et al., 2012).

#### Wagnild and Young’s Resilience Scale-Short Version

The Resilience Scale of 25 items, which measures the capacity to bear life stressors that have rated on a 7-point Likert-type developed by Wagnild and Young (1993), was adapted into Turkish as 24 items by Terzi (2006). This scale consists of two factors: Self-Efficacy and Acceptance of Self and Life. Recently, Işık et al. (2019) revised it to obtain a short form of the scale. The short form has 10 items that is rated on a 7-point Likert-type (from 1 = “*Absolutely Disagree*” to 7 = “*Absolutely Agree*”) and has a single-factor structure and measures a similar psychological concept. The short version of RS has good validity and reliability (Işık et al., 2019). In this study, the Cronbach’s alpha for the scale was 0.90.

#### Trait Emotional Intelligence Questionnaire—Short Form

According to Furnham and Petrides (2003), emotional intelligence is a personality trait related to how emotionally efficient an individual feels. The current full form of the scale, which was first developed in 2001, consists of 4 factors, 15 facets, and 153 items (Petrides, 2009). The short form includes 30 items in a 7-point Likert-type, which covers the trait EI factors: emotionality, self-control, sociability, and wellbeing (Petrides and Furnham, 2003). The Turkish version of the short form has a structure consisting of 20 items with 4 factors, which is responded to on a 7-point Likert scale (from 1 = “*Completely Disagree*” to 7 = “*Completely Agree*”) (Deniz et al., 2013). The reliability of the total scale in the original study was “g”; alpha = 0.73 (Furnham and Petrides, 2003), in the Turkish adaptation study, Cronbach’s alpha was 0.81 for the total scale and the four subscales showed appropriate Cronbach’s alphas ranging between 0.72 (wellbeing) and 0.66 (emotionality) (Deniz et al., 2013). Similarly, in this study, Cronbach’s alpha was 0.83 for the total scale and the four subscales showed appropriate Cronbach’s alphas ranging from 0.67 (wellbeing) and 0.62 (emotionality).

In the three previous studies using TEIQue Short Form in university students, the mean values of global emotional intelligence score were in the range between 92.21 and 97.81. The mean values for wellbeing, self-control, emotionality, and sociability were 19.97–21.94, 17.49–21.01, 18.81–19.93, and 19.29–20.2, respectively (Özer and Deniz, 2014; Bağdiken, 2021; Yağcan et al., 2021).

### Statistical Analyses

In the study, a latent profile analysis was conducted to reveal the latent profiles of participants according to their responses to SSI-R. Latent profile analysis is a statistical method used to reveal unobserved subgroups in a population through a set of continuous variables. Instead of *k*-means method of the nonhierarchical cluster analysis methods, a latent profile analysis, which is included in the finite mixture models, was preferred. Finite mixture models represent a mixture or composite of the overall distribution of one or more variables, a finite number of components’ (components cannot be observed directly) distributions. Population heterogeneity in a set of observed variables is assumed to result from two or more homogeneous subgroups that are separated from each other (Masyn, 2013). Finite mixture models make clustering based on a probability-based model describing the distribution of the data, while a clustering analysis makes clustering based on an arbitrarily chosen distance measurement (Nylund et al., 2007). A probabilistic clustering approach assumes that each object/case belongs to a class or cluster but takes into account the uncertainty of the object/case’s class membership. Although this makes the latent class analysis conceptually similar to fuzzy clustering methods, whereas in the fuzzy clustering method the class membership of the object/case is the estimated “parameters,” in the latent class analysis the probability of an individual’s posterior class-membership probabilities are computed from the estimated model parameters and his/her observed scores. This calculation makes it possible to classify other objects/cases in the population from which the sample has been taken. This is not possible with standard fuzzy clustering techniques. Another advantage of model-based clustering is that it does not require making decisions about the scaling of the observed variables, for example, when working with a normal distribution of unknown variances, standardizing the variables will not affect the result. However, scaling is always an issue in nonhierarchical clustering methods. In addition to analyzing the variables measured at different scale types, model-based clustering has other advantages such as deciding on the number of clusters and other model features through more formal criteria (Vermunt and Magidson, 2002, pp. 89–91).

In this study, the latent profile analysis was performed by R (version 4.1.0, R Core Team, 2021) running in Rstudio (version 1.4.1106 used here). There are many R packages with latent class/mixture analytical functionality, but most of these packages focus on analyses with discrete indicator variables or require a lot of coding to define the needed models. Therefore, the more practical “mclust” (Scrucca et al., 2016) and “tidyLPA” (Rosenberg et al., 2018) packages were used. Deciding of how many classes best describe the patterns observed in the data in an latent profile analysis is similar to deciding on the number of factors to retain in an exploratory factor analysis. There is not a single fit index but a set of statistical fit indices. Information criteria, likelihood-based tests and entropy index are among these fit indices. Lower values of information criteria, such as Bayesian Information Criterion (BIC), Sample Size Adjusted Bayesian Information Criterion (SABIC), Akaike Information Criterion (AIC), Consistent Akaike Information Criterion (CAIC), and Integrated Completed Likelihood Criterion (ICL), indicate a better fit of the model. Likelihood-based tests, such as the BLRT, allow the use of the value of *p* to compare the models with *k* and *k*-1 classes. A non-significant value of *p* provides support for the *k*-1 class model vs. the *k*-class model. Thus, it is tested whether there is a statistically significant improvement in model fit by increasing the number of classes (Nylund et al., 2007). The entropy index is an indicator of how well individuals are classified in the model. Values more than 0.80 indicate “good” classification (Clark and Muthén, 2009). In all models, the cluster-specific mean of each *k*-class is estimated. Each class has its own pattern of mean scores on indicator variables to determine profiles. Model variants are distinguished from each other according to whether a covariance matrix of the indicator variables is allowed to vary within and between the classes (Celeux and Govaert, 1995; Scrucca et al., 2016). The “mclust” package was used to determine an optimal model variant. The best model variant was that the covariances within and between the classes in the model was 0, the variances of the indicator variable vary within the classes but equal between the classes. Because only a set of variances need to be estimated, this model has been expressed as the most parsimonious model type (Wardenaar, 2021).

After determining the model, to test group differences, the one-way ANOVA was used to compare the differences in the stressors, reactions to stress, psychological resilience, and emotional intelligence between the identified stress profiles. *Post*-*hoc* tests were performed to identify which groups had statistically significant differences between them. The effect sizes were calculated using the eta-squared index (^***^n^2^) to obtain the magnitude of the observed differences. The ^***^n^2^ index was interpreted as follows: the values between 0.0099 and 0.0588 indicated a small effect size; the values between 0.0588 and 0.1379 a medium effect size; and the values more than 0.1379 a large effect size (Cohen, 1988, pp. 285–288).

## Results

### Preliminary Analyses

Internal consistency of the scales, descriptive statistics and correlations between the Student-Life Stress Inventory and its nine categories, resilience, emotional intelligence, and its four factors are presented in Table 1.

**TABLE 1:** Descriptive statistics and correlations between the study variables.

	TS	F	CON	P	CHG	SI	PR	ER	BR	CR	R	EI	WB	SC	E	S
**TS**																
F	0.77*															
CON	0.63*	0.52*														
P	0.79*	0.63*	0.42*													
CHG	0.67*	0.58*	0.44*	0.58*												
SI	0.67*	0.47*	0.40*	0.57*	0.38*											
PR	0.85*	0.50*	0.41*	0.57*	0.46*	0.48*										
ER	0.82*	0.55*	0.47*	0.63*	0.49*	0.53*	0.65*									
BR	0.77*	0.55*	0.42*	0.52*	0.47*	0.43*	0.57*	0.65*								
CR	–0.18*	–0.13^b^	–0.09	–0.12^b^	–0.00	0.00	–0.07	–0.16^b^	–0.02							
**R**	–0.36*	–0.30*	–0.27*	–0.28*	–0.22*	–0.03	–0.27*	–0.32*	–0.24*	0.41*						
**EI**	–0.55*	–0.50*	–0.39*	–0.46*	–0.37*	–0.23*	–0.39*	–0.48*	–0.40*	0.37*	0.64*					
WB	–0.34*	–0.36*	–0.23*	–0.28*	–0.26*	–0.09	–0.20*	–0.29*	–0.27*	0.35*	0.69*	0.57*				
SC	–0.66*	–0.54*	–0.55*	–0.47*	–0.48*	–0.37*	–0.46*	–0.54*	–0.47*	0.33*	0.47*	0.61*	0.42*			
E	–0.33*	–0.31*	–0.27*	–0.20*	–0.26*	–0.20*	–0.24*	–0.21*	–0.25*	0.14^b^	0.21*	0.39*	0.22*	0.37*		
S	−0.32*	–0.27*	–0.23*	–0.27*	–0.12^b^	–0.08	–0.28*	–0.29*	–0.19*	0.26*	0.55*	0.56*	0.34*	0.40*	0.33*	
$\bar{x}$	151.42	20.77	12.46	13.00	8.44	20.25	33.69	13.88	19.84	8.91	51.51	88.91	19.04	16.41	17.57	18.62
*sd*	32.05	5.00	3.88	3.96	3.08	3.86	11.30	4.05	6.01	2.77	10.86	16.80	4.47	4.75	4.25	4.76
α	0.94	0.76	0.86	0.78	0.84	0.61	0.86	0.81	0.74	0.80	0.90	0.83	0.67	0.64	0.62	0.64

TS = total stress; F = frustrations; CON = conflicts; P = pressures; CHG = changes; SI = self-imposed; PR = physiological reactions; ER = emotional reactions; BR = behavioral reactions; CR = cognitive reactions; R = psychological resilience; EI = emotional intelligence; WB = well-being; SC = self-control; E = emotionality; S = sociability. *p < 0.001, ^b^p < 0.01.

Correlations between emotional intelligence and the stressors, reactions to stressors, and psychological resilience were statistically significant and moderate in most cases. Psychological resilience was moderately negatively correlated with total stress. While resilience and EI were negatively correlated with reactions to stressors (except cognitive reactions), they were moderately positively correlated with cognitive reactions. Whereas EI was highly negatively correlated with total stress, it was highly positively correlated with resilience. The mean values of trait emotional intelligence and its four factors in this study were slightly lower compared to previous studies (see Özer and Deniz, 2014; Bağdiken, 2021; Yağcan et al., 2021).

### Stress Profiles

Model fit for solutions with 1–9 latent classes were examined (see Table 2). With an increase in the number of classes, the log likelihood and AIC values constantly decreased, and the BLRT indicated significant results in comparisons, which are all the models with *k* and *k*-1 classes. Thus, these values did not suggest any solution. In the 9-class model solution, the R program indicated that less than 1% (4 people) of the participants take part in one of the classes, and recommended that the other models were examined. It is seen that the SABIC value suggested to the 8-class solution, while the BIC, CAIC, and ICL values suggested to the 5-class model solution, and the entropy index was 0.95. Allocating each subject in a single class with sufficient certainty is necessary to obtain a useful classification. In this study, the average latent class-membership probabilities were in the range from 0.96 to 0.98 in the 5-class model solution. In the selection of the final model, in addition to these indicators, the principle of parsimony and the meaningfulness and usefulness of the model should also be considered (Masyn, 2013). Thus, it was concluded that the 5-class model was the model that best fits the data.

**TABLE 2:** Latent profile analysis of stress: Statistical fit indices.

Model	*BIC*	*CAIC*	*ICL*	*AIC*	*SABIC*	*LL*	*Entropi*	p_BLRT_
1	72334.96	72440.96	–72334.96	71907.20	71998.59	–35847.60	1.00	
2	68878.18	69038.18	–68897.39	68232.50	68370.46	–33956.25	0.94	0.01
3	68004.71	68218.71	–68029.44	67141.12	67325.63	–33356.56	0.95	0.01
4	67871.88	68139.88	–67900.14	66790.37	67021.44	–33127.19	0.95	0.01
5	67597.52	67919.52	–67627.90	66298.09	66575.72	–32827.05	0.95	0.01
6	67649.50	68025.50	–67681.99	66132.16	66456.35	–32690.08	0.95	0.01
7	67633.98	68063.98	–67671.69	65898.72	66269.47	–32519.36	0.95	0.01
8	67705.97	68189.97	–67744.34	65752.80	66170.11	–32392.40	0.95	0.01
9	67914.88	68452.88	–67950.49	65743.79	66207.65	–32333.89	0.96	0.01

*AIC = Akaike Information Criterion; BIC = Bayesian Information Criterion; CAIC = Consistent Akaike Information Criterion; ICL = Integrated Classification Likelihood; LL = Log Likelihood; SABIC = Sample-Size Adjusted Bayesian Information Criterion; p_BLRT_ = p-value of the bootstrapped likelihood ratio test for k versus k-1 classes.*

The total stress scores of the groups were in the range from 101.55 to 202.31. In the seven previous studies, the total stress score was in the range between 109.6 and 177.6 (see Gadzella, 2004; Baloğlu and Bardakcı, 2010; Gadzella et al., 2012). The group with the lowest average stress score was defined as an “extremely low stress group (ELSG),” and the group with the highest average stress score was defined as an “extremely high stress group (EHSG).” The group with a total stress score higher than ELSG and lower than Class 3 was defined as a “low stress group (LSG).” The group with a total stress score lower than EHSG and higher than Class 3 was defined as a “high stress group (HSG).” The group with a total stress score lower than HSG and higher than LSG was defined as a “medium stress group (MSG).” Extremely low stress group contained 60 (14%), LSG 131 (31%), MSG 134 (32%), HSG 38 (10%), and EHSG 55 (13%) individuals (see Table 3).

**TABLE 3:** The differences of five classes in terms of the study variables: Descriptive statistics, results of ANOVAs, and *post hoc* comparisons.

Variables	Class 1 (ELSG; *n* = 60)		Class 2 (LSG; *n* = 131)		Class 3 (MSG; *n* = 134)		Class 4 (HSG; *n* = 38)		Class 5 (EHSG; *n* = 55)		ANOVA (4, 413)		*Post hoc* comparisons
	$\bar{x}$	ss	$\bar{x}$	ss	$\bar{x}$	*ss*	$\bar{x}$	ss	$\bar{x}$	ss	F	η^2^	
Total stress^a^	101.55	11.39	133.97	10.36	163.93	11.76	172.55	13.86	202.31	14.71	648.85*	0.91	5 > 4 > 3 > 2 > 1
Frustrations^a^	15.02	3.41	18.77	3.23	22.63	3.82	21.18	4.02	27.02	3.57	100.49*	0.46	5 > 3,4; 4 > 2; 3 > 2 > 1
Conflicts^a^	7.53	2.24	11.98	3.05	13.43	3.22	13.03	3.14	16.22	3.43	63.84*	0.34	5 > 3,4; 4 > 1; 3 > 2 > 1
Pressures^a^	7.92	2.34	11.10	2.68	15.01	2.55	13.11	2.30	18.09	2.02	162.57*	0.55	5 > 4,3; 3 > 4; 4 > 2; 3 > 2 > 1
Changes^a^	5.52	2.26	7.09	2.13	9.43	2.52	8.53	2.37	12.33	2.32	79.29*	0.40	5 > 3,4; 4 > 2; 3 > 2 > 1
Self-imposed^a^	16.60	3.89	18.70	3.19	21.63	2.80	21.32	3.37	23.78	3.09	52.07*	0.34	5 > 3, 4; 4 > 2; 3 > 2 > 1
Physiological reactions^b^	20.37	3.65	28.24	6.12	34.09	6.11	48.00	6.63	50.35	7.93	248.44*	0.75	5,4 > 3 > 2 > 1
Emotional reactions^a^	7.78	1.96	11.95	2.37	16.09	2.39	15.53	2.53	18.62	1.86	227.22*	0.62	5 > 3, 4; 4 > 2; 3 > 2 > 1
Behavioral reactions^b^	12.38	3.07	17.31	3.90	21.94	4.20	24.45	6.06	25.73	5.01	101.81*	0.48	5 > 3 > 2 > 1; 4 > 2 > 1
Cognitive reactions^b^	9.57	3.22	9.18	2.53	8.33	2.53	10.58	2.13	7.82	2.95	8.86*	0.05	1, 2 > 5; 4 > 2, 3, 5
Resilience^a^	58.40	8.51	53.98	9.19	48.84	10.32	50.32	10.65	45.51	12.87	16.10*	0.23	1, 2 > 3,5; 1 > 4
Emotional intelligence^a^	105.22	13.86	94.25	12.76	84.01	13.92	86.26	13.79	72.25	16.02	50.07*	0.43	1 > 2 > 3, 4 > 5
Well-being^a^	21.82	3.85	19.68	3.50	18.15	4.21	19.29	4.39	16.47	5.79	13.89*	0.16	1 > 2 > 3; 1 > 4; 2 > 5
Self-control^a^	21.15	3.38	18.31	3.72	14.83	3.90	15.66	3.50	11.09	3.69	67.70*	0.41	1 > 2 > 3; 2 > 4 > 5; 3 > 5
Emotionality^a^	19.88	3.86	17.72	3.95	17.42	4.10	17.11	4.18	15.42	4.58	8.80*	0.11	1 > 2, 3, 4; 2 > 5
Sociability^a^	21.03	4.77	19.78	4.10	17.63	4.91	17.13	4.58	16.65	4.29	11.58*	0.11	1 > 3, 4, 5; 2 > 3, 4, 5

*p < 0.001. ^a^ Scheffe test for post hoc comparisons was used because the variable satisfied the assumption of homoscedasticity. ^b^ Tamhane’s T2 test for post hoc comparisons was used because the variable violated the assumption of homoscedasticity.

Extremely low stress group has scored lower levels of stressors, reactions to stressors (except cognitive reactions) compared to the other groups with large effect sizes. Similarly, LSG has scored lower levels of stressors, reactions to stressors (except cognitive reactions) compared to the groups with stress higher than itself. ELSG has scored higher levels of emotional intelligence, wellbeing, self-control, and emotionality compared to the other groups with large and medium effect sizes. LSG showed higher scores of emotional intelligence, wellbeing, self-control, and emotionality compared to the groups with stress higher than itself. ELSG and LSG have scored higher on the sociability factor than MSG, HSG, and EHSG. On the contrary, EHSG showed higher scores of stressors, reactions to stressors (except cognitive and physiological reactions) compared to other groups with large effect sizes. Nonetheless, EHSG has scored lower levels of emotional intelligence, and self-control compared to other groups with large effect sizes.

In terms of cognitive reactions, EHSG has scored lower than ELSG, LSG, and HSG. When it comes to MSG and HSG, they differed only with regard to pressures and physiological and cognitive reactions. Spectacularly, whereas MSG has felt more pressure than HSG, HSG has scored significantly higher physiological and cognitive reactions compared to MSG and LSG. In regard to psychological resilience, ELSG has scored higher than MSG, HSG, and EHSG but did not differ significantly from LSG. LSG has scored higher resilience than MSG and EHSG but did not differ significantly from HSG.

**FIGURE 1:**
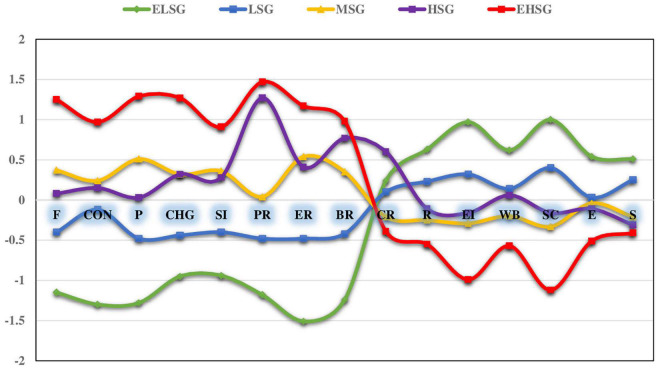
Standardized means of study variables for stress profiles. F, frustrations; CON, conflicts; P, pressures; CHG, changes; SI, self-imposed; PR, physiological reac.; ER, emotional reac.; BR, behavioral reac.; CR, cognitive reac.; R, resilience; EI, emotional intelligence; WB, well-being; SC, self-control; E, emotionality; S, sociability.

The trait emotional intelligence scores of ELSG (0.97 SD) and LSG were higher than the average, while the MSG, HSG, and EHSG (− 0.99 SD) were lower than the average. Similarly, the resilience scores of ELSG (0.63 SD) and LSG were higher than the average, while the MSG, HSG, and EHSG (− 0.55 SD) were lower than the average (see Figure 1).

## Discussion

As a result of the analysis for RQ1, we explored that five groups were differentiated from each other with a large effect size. Nearly half of the students (about 45%) are in ELSG and LSG. This finding may indicate a positive change in students’ level of stress as it was obtained after the first wave of the COVID-19 pandemic (see Goppert and Pfost, 2021). Benham (2020) reported that, while there was no significant difference in the level of stress between Spring 2019 and Spring 2020, there was a significant decrease in the level of stress between Spring 2020 and Summer 2020 in a longitudinal study, which he examined the stress of college students. The fact that students did not have to get up early to go to university for classes and could watch the recorded lectures whenever they want gave them more flexibility in planning their time. Flexibility in planning time and longer sleep durations (Benham, 2020) may have had a positive effect on students’ stress.

As a result of the analysis for RQ2 and RQ3, ELSG, LSG, MSG, and EHSG differ significantly in terms of stressors and most of the reactions to stressors. MSG and HSG significantly differed only in regard to pressures and physiological and cognitive reactions. The level of physiological reactions was highly positively correlated with the stress. The frequencies of physiological reactions of HSG and of those of EHSG are similar. However, interestingly, cognitive reactions of HSG have a significantly higher frequency than those of the other groups except ELSG. Cognitive reactions involve the individual’s evaluations about a situation in which one finds oneself. If there is a stressful situation, it helps a person to decide whether she/he has sufficient resources and effective strategies to cope with the situation (Lazarus and Folkman, 1984). Despite its high frequency of cognitive reactions, HSG has scored a high level of stress possibly because the students in this group see a high level of stress as an inevitable part of their lives, regardless of the resources they have or the effectiveness of their coping strategies. Also, HSG has scored a high level of physiological responses to stressors, similar to EHSG. There was a positive and significant relationship between the level of stress and physiological reactions. They seem to have compensated for a high level of stress that they have perceived through somatization (see Hu and Shao, 2016; Shangguan et al., 2021). Furthermore, standards that MSG, HSG and EHSG have imposed on themselves are higher than ELSG and LSG. As noted by Frost et al. (1990), striving to achieve high standards is not pathological in itself. Psychological problems are related to perfectionism, which employs overly critical self-evaluations (p. 450). It is possible that intense somatization experienced by HSG and EHSG is related to negative self-evaluations (Dunkley et al., 2006, p. 65). This relationship has been confirmed by some studies in the literature (Nussbaum and Goreczny, 1995; Hollifield et al., 1999).

In parallel with the previous studies, the relationship between emotional intelligence and stress was found to be negative (Enns et al., 2018; Trigueros et al., 2020; Mérida-López and Extremera, 2021), a significant negative relationship was also found between emotionality (emotion perception and expression) and stress (Ramesar et al., 2009; Salguero et al., 2011; Finlay-Jones et al., 2015). The fact that there were significant differences in emotionality factor in favor of ELSG compared to other groups and in favor of LSG compared to EHSG, which supports the argument that there is a relationship between the capacity to perceive and express one’s own feelings and the level of stress. Also, it is seen that emotionality scores decreased as the level of stress increased in other groups, but there was no significant difference. Some studies reported that there is no significant relationship between emotionality/emotion perception and stress (Ciarrochi et al., 2002; Arora et al., 2011). However, in the study by Ciarrochi et al. (2002), although there was no direct relationship between emotion perception and stress, depression, suicidal thoughts, and hopelessness, a group with high emotion perception had significantly higher levels of depression, hopelessness, and suicidal thoughts rather than a low emotion perception group (Schwab and Schienle, 2017; see Gutiérrez-García and Calvo, 2017; Cui et al., 2021). They stated that emotion perception is a moderator variable between stress and mental disorders. This may be because some people with high emotion perception maladaptively focus on their negative emotions. However, because the emotionality factor used in this study includes the ability to express one’s emotions as well as being aware of one’s own emotions, it is possible to make it easier for the person to meet one’s own needs and thus enable one to cope with stress effectively.

In the sociability factor, which includes managing the emotions of others, ELSG and LSG have significantly higher mean scores than the more stressed groups. In line with the findings of previous studies, sociability and the level of stress were negatively correlated (Penley and Tomaka, 2002; Ramesar et al., 2009; Arora et al., 2011). As the level of stress increased, sociability scores decreased, but there was no significant difference in other groups. People who activate the positive moods of others often access a more social support (Ciarrochi et al., 2002, p. 199). Social support plays a critical role in a person’s resilience and wellbeing (Yıldırım and Tanrıverdi, 2021). Although sociability is a protective factor against stress, the inclusion of other factors in the formula for coping effectively with stress seems necessary to understand stress groups.

Emotional and behavioral reactions were highly positively correlated to the level of stress and moderately negatively correlated to self-control. The emotion regulation process enables a person to manage stress by regulating one’s own emotional state, the importance of the event/situation one is facing, and the behavioral expression of emotion (sometimes by preventing reactions) (Eisenberg et al., 2007, p. 288). The level of stress trended to decrease as groups’ level of self-control trended to increase (Finlay-Jones et al., 2015; Mc Gee et al., 2018; Siddiqui et al., 2021).

As the level of stress trended to increase, the level of wellbeing trended to decrease. However, there was no significant difference in the levels of wellbeing of MSG, HSG, and EHSG. The similarity between psychological resilience and emotional intelligence of HSG to MSG may prevent a significant negative effect of stress on the wellbeing and functionality of the HSG. As the frequency of exposure to stressors increased, the level of stress trended to increase. Exceptionally, more frequent pressures were reported in MSG than HSG. People in MSG may find the situations in which they are under pressure unpleasant and threatening compared to those in HSG. However, pressures such as “competition, meeting deadlines, trying to do too many things at once” may be acting to improve performance for HSG (see Ferrari et al., 2009; Bell et al., 2012; Grunschel et al., 2013).

A negative significant relationship was found between the level of stress and psychological resilience (Hou et al., 2017; Zhang et al., 2018; Shangguan et al., 2021; Traunmüller et al., 2021; Yalcin-Siedentopf et al., 2021). However, ELSG and LSG had higher psychological resilience than MSG and EHSG, whereas MSG, HSG, and EHSG did not differ significantly from each other. And, ELSG had higher psychological resilience than HSG, whereas ELSG and LSG did not differ significantly from each other. The resilience of HSG is similar to MSG, which may be due to the fact that the HSG, who experiences high levels of stress, struggles to become more resilient (see Nishi et al., 2010; Um et al., 2014). Fishman (2012) reported that the relationship between resilience and perceived stress of university students was found not to be statistically significant. Similarly, Bitsika et al. (2013) found no significant direct interaction between daily stress and resilience. The study was conducted on parents of a child with an Autism Spectrum Disorder. Parents with high daily stress had significantly higher anxiety and depression than parents with low daily stress. Parents whose resilience scores were low also showed significantly higher anxiety and depression than parents whose resilience scores were high. Resilience did appear to buffer against anxiety and depression, ensuring that parents could continue to meet their children’s emotional and physical needs, no matter how stressed they were. Resilience seems to take on the function of preventing intense stress from turning into anxiety and depression in the long run. We can say that stress groups have the capacity to cope with stress in some ways, regardless of the level of stress they experience. Due to the cross-sectional nature of this study, possible divergences between the groups over time could not be observed. A longitudinal study may be useful to observe possible divergences.

The study is limited to university students in Turkey, which may affect the generalizability of this research. Because other factors such as some other personality traits and types (hedonist, spectator, sceptic, openness to experience, conscientiousness, neuroticism, agreeableness, etc.), symptoms (depression, interpersonal sensitivity, etc.) were not taken into account, an understanding of memberships of stress groups remained limited. A lot of studies on stress and personality traits, other than resilience and trait EI, indicate significant correlations between them (see Tyssen et al., 2007; Afshar et al., 2015; Garbe et al., 2020; Liu et al., 2021). Furthermore, some longitudinal studies have reported counterintuitive findings on the relationship between them (see Yap et al., 2012; Anusic et al., 2014; Hahn et al., 2015; Mitchell et al., 2021). In future studies, stress groups can be investigated in regard to these factors. Because our search for stress groups was exploratory, it is necessary to examine its validity through confirmatory analyses in future studies. By conducting semi-structured interviews with people in different stress groups, a deeper understanding of their stress perceptions (e.g., state and trait), stress types, and reactions can be revealed.

## Conclusion

We conclude that the COVID-19 pandemic might not have linked to greater perceived stress, and university students may have become resilient to the changes in their lifestyles due to the pandemic. Our results showed that (1) although some stress groups differed from each other with a large effect size in terms of stress, they did not show significant differences with regard to emotional intelligence and resilience (see MSG and HSG); (2) Despite experiencing high levels of stress, some students (see HSG) reported that they were able to effectively cope with stress. Although these students experience more stress than most of others, they reported that they experience low pressure such as “competition, meeting deadlines, trying to do too many things at once.” This finding necessitates a study on how students perceive the type of stress they experience (threatening or challenging); (3) Students with a lower level of stress than the average reported higher levels of emotional intelligence (wellbeing, self-control, and emotionality) and resilience than others. Students with the highest level of stress reported lower levels of emotional intelligence (specifically self-control) than others; (4) Emotional, physiological, and behavioral reactions were highly positively correlated to the level of stress and moderately negatively correlated to self-control; (5) Whereas resilience was highly positively related to wellbeing, resilience and wellbeing were moderately negatively related to stress. However, because MSG, HSG, and EHSG did not differ in regard to resilience and wellbeing, it is shown that they maintain their functionality by coping with stress in some ways, no matter how stressful they are. In future, a longitudinal study can reveal possible divergences that may occur in stress groups. The design of interventions for stress groups will be facilitated.

## Data Availability Statement

The raw data supporting the conclusions of this article will be made available by the authors, without undue reservation.

## Ethics Statement

The studies involving human participants were reviewed and approved by the Scientific Research and Publication Ethics Committee of Social and Human Sciences of Usak University (ref no. 2021-122). All participants were informed about the procedure and provided their electronic consent.

## Author Contributions

All listed authors have made a substantial, a direct, and an intellectual contribution to this work, and approved it for publication.

## Conflict of Interest

The authors declare that the research was conducted in the absence of any commercial or financial relationships that could be construed as a potential conflict of interest.

## Publisher’s Note

All claims expressed in this article are solely those of the authors and do not necessarily represent those of their affiliated organizations, or those of the publisher, the editors and the reviewers. Any product that may be evaluated in this article, or claim that may be made by its manufacturer, is not guaranteed or endorsed by the publisher.
